# A Rough Energy Landscape to Describe Surface-Linked Antibody and Antigen Bond Formation

**DOI:** 10.1038/srep35193

**Published:** 2016-10-12

**Authors:** Laurent Limozin, Pierre Bongrand, Philippe Robert

**Affiliations:** 1 Laboratoire Adhesion & Inflammation, UMR INSERM 1067, UMR CNRS 7333, Aix-Marseille Université, Assistance Publique-Hôpitaux de Marseille, Case 937, 13288 Marseille Cedex 09, France

## Abstract

Antibodies and B cell receptors often bind their antigen at cell-cell interface while both molecular species are surface-bound, which impacts bond kinetics and function. Despite the description of complex energy landscapes for dissociation kinetics which may also result in significantly different association kinetics, surface-bound molecule (2D) association kinetics usually remain described by an on-rate due to crossing of a single free energy barrier, and few experimental works have measured association kinetics under conditions implying force and two-dimensional relative ligand-receptor motion. We use a new laminar flow chamber to measure 2D bond formation with systematic variation of the distribution of encounter durations between antigen and antibody, in a range from 0.1 to 10 ms. Under physiologically relevant forces, 2D association is 100-fold slower than 3D association as studied by surface plasmon resonance assays. Supported by brownian dynamics simulations, our results show that a minimal encounter duration is required for 2D association; an energy landscape featuring a rough initial part might be a reasonable way of accounting for this. By systematically varying the temperature of our experiments, we evaluate roughness at 2*k*_*B*_*T*, in the range of previously proposed rough parts of landscapes models during dissociation.

Ligand-receptor interactions have long been described with the formalism elaborated by chemists for reactions in solution, i.e., affinity at equilibrium, and kinetics by on-rate and off-rates. However, ligand-receptor interactions occurring at cell-cell interfaces may differ significantly from what happens in solution. First, forces may be applied to the interaction either directly (e.g. via molecular motors) or indirectly (e.g. via hydrodynamic forces). Second, relative ligand-receptor motion is bi dimensional (2D) instead of tri dimensional (3D), being limited to the membrane plane, with further confinement arising from objects such as membrane domains or cytoskeleton. Such alterations in transport should strongly affect kinetics of bond formation^1^,^2^. For example, the kinetics of the B Cell Receptor (BCR, structurally identical to an antibody linked to a B-cell surface) interaction with antigen may differ strongly from the kinetics of antibody-antigen interaction in solution (as was already shown for the similar T Cell Receptor-Major Histocompatibility Complex bound peptide (TCR-pMHC) interaction^3^,^4^,^5^,^6^). Both BCR-antigen bond formation and bond rupture occur in 2D conditions, and are critical for B cell activation during the immune response. First, most of the antigen detected in a lymph node by B lymphocytes prior to their activation is not in soluble form but linked to resident macrophages or dendritic cells^7^,^8^,^9^. B lymphocytes make ≪endocytic synapses≫ with these cells^10^,^11^, and indeed the B lymphocyte was shown to pull on its BCR, this pulling being critical to ligand discrimination^12^. Second, during somatic hypermutation (which may follow B lymphocyte activation and where several mutation-selection cycles in the lymph node lead to a strong increase in antibody affinity, from 10^4^M up to 10^10^M^13^,^14^) B cells also do probe their ligand by exerting a force on the BCR-antigen bond. This pulling phase is also of considerable importance for ligand discrimination^12^,^15^. In addition, association kinetics of the BCR-antigen bond (and resulting antibody-antigen bond) could be specifically modified during affinity maturation. Foote and Milstein early described an increase in on-rate^16^, which was recently confirmed in another model^17^,^18^.

The effect of a disruptive force on off-rate has been measured for numerous molecular interactions at the single molecular level, and plays a direct physiological role in interactions such as selectin-PSGL1^19^,^20^ and TCR-pMHC^3^,^4^,^5^, thus emphasizing the need for such measurements. However, on-rate measurements in 2D remain scarce and could benefit from further exploration^4^,^21^. Indeed, while the relationship between bond rupture and complex energy landscapes describing molecular interaction has been extensively studied^22^, the description of bond formation is still based on on-rates, corresponding to one free energy barrier (Δ*E*_*A*_) leading to one free energy well (see Fig. 1a). Probability of bond formation as a function of the duration *t*_*e*_ during which a receptor interacts with its ligand (referred later as “encounter duration”) can be written as


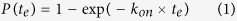


where *k*_*on*_ is the on-rate. Recently, we observed discrepancies between bond formation measurements performed with the laminar flow chamber and the on-rate model. Probability of bond formation was not proportional to encounter duration: we proposed a bond formation model^23^,^24^ based on a rough initial part in the energy landscape (the rough energy landscape being a concept first suggested by Zwanzig^25^ in another context). In this model, the first part of the energy landscape is made of numerous small energy peaks (forming the rough part of the landscape, of length *l* and roughness *ε*) before a free energy well (see Fig. 1b). Bond formation results from crossing the rough part of the landscape; this crossing occurs as a very slow diffusion process^23^,^24^. Probability of bond formation was shown to match the following simple law:


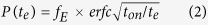


where *f*_*E*_ is a phenomenological factor assumed to represent the proportion of properly folded and functional molecules, *erfc* is the complementary error function, and *t*_*on*_ is a characteristic time of the bond. From a theoretical point a view, recent reports suggest that binding kinetics of membrane attached molecules can be recalculated by accounting for membrane fluctuation and roughness^26^,^27^,^28^. However, the molecular intrinsic association rate is not questioned in these studies.

In a laminar flow chamber, receptor-coated microspheres move in a shear flow on top of a surface bearing ligand molecules. If a receptor binds its ligand, the microsphere stops, while a force is immediately applied to the bond. During an experiment at a given shear rate, the number of association events and the total distance travelled by microspheres after sedimentation are measured, their ratio being called “binding linear density” (in μm^−1^). A first simulation work follows to describe the microspheres and ligand and receptor movements responsible for bringing ligand and receptor together prior to their interaction, thus calculating the distribution of the durations during which one ligand may interact with one receptor (or “encounter durations”) for the experimental condition. A second simulation work uses binding models to retrieve simulated binding linear density, and permits comparison of these models to the experimental binding linear density^24^,^29^,^30^. The distribution of durations during which one ligand may interact with one receptor (or “encounter durations”) is essential for calculation of kinetic rates^30^,^31^. In assays where one of the reactants is in solution such as surface plasmon resonance, this distribution depends solely on diffusion. This distribution is directly controlled in a laminar flow chamber, usually by varying the shear rate^23^. In the present study, we added two innovative features to the laminar flow chamber: first, the distance between microsphere and surface was varied by tilting the set-up (see Fig. 2a,b). This changed the distribution of encounter durations independently of shear, thus independently of applied force. This allowed us to obtain a large number of experimental conditions, differing either by shear rate or average microsphere distance to the surface, that were fitted for each binding model with the same set of parameter. This permitted to compare the validity of each binding model, and supported at the same time the validity of the model of microsphere and molecular movement. Second, temperature was controlled and systematically varied to obtain quantitative information on the thermodynamics of the process. Besides, to measure kinetics, it is necessary to collect a large number of individual association and dissociation events due to their stochastic nature. We built a new automated laminar flow chamber set-up in order to maximize data acquisition, used to measure the association and dissociation kinetics of a model antibody-antigen system at the single molecular level. We systematically varied shear rate and tilt angle to put our numerical models to test and to compare two alternative models of binding kinetics. One model was based on one free energy barrier, giving a classical on-rate (*k*_*on*_), the second model was based on an energy landscape featuring a rough initial part, giving a minimum encounter time model (*t*_*on*_). We show that 2D association kinetics strongly differs from 3D association kinetics measured using surface plasmon resonance, and that a rough energy landscape resulting in a minimal encounter time may be more suitable than an on-rate to describe association kinetic. By varying the temperature, we described more precisely the energy landscape, as we estimated the roughness of its initial part.

## Results

### Surface plasmon resonance measurements

Surface plasmon resonance was used to measure the kinetics of this antibody-antigen bond in soluble form (see Supplementary Fig. S1). Incremental amounts of soluble anti-HLA were incubated on pMHC coated surfaces and the SPR response was monitored with time. On-rate (*k*_*on*_) was determined by fitting directly the kinetics of the surface plasmon resonance signal, using standard equation implemented in surface plasmon resonance analysis software. As off-rate (*k*_*off*_) was low, its measurement by kinetics of the surface plasmon resonance signal was feared imprecise, so affinity (*K*_*D*_) was measured at equilibrium and off-rate calculated as


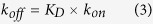


Results were as follows: *k*_*on*_ = 1.4 ± 0.3 × 10^5^*M*^−1^*s*^−1^, *K*_*D*_ = 1 ± 0.3 × 10^−8^*M*, calculated *k*_*off*_ = 1.4 × 10^−3^*s*^−1^.

### Evidencing single molecular association under 2D conditions

Single bond measurements were performed using the usual method for laminar flow chamber experiments^5^. Flow chamber experiments were performed on substrates coated either without ligand as a negative control, or coated with seven different amounts of ligand, doubling from one condition to the next, thus varying relatively from one to sixty-four (incubation concentrations were varied from 0.0025 μg/ml to 0.16 μg/ml). Experiments were repeated on average 7 times per density condition; 6 shear rate conditions were applied for each density condition, from 20*s*^−1^ to 120*s*^−1^. Force on bond was calculated as


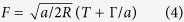


with


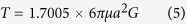


and


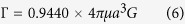


(with *T* the hydrodynamic traction on the microsphere, Γ the torque on the microsphere, *a* the microsphere diameter (4.5 μm), *R* the total bond length (24 nm), *μ* the medium viscosity (10^−3^ Pa.s), and *G* the shear rate)^32^, exerting hydrodynamic forces on the bonds from 38pN to 228pN respectively. For the four lowest amounts of ligand (0.0025 μg/ml, 0.005 μg/ml, 0.01 μg/ml and 0.02 μg/ml, forming an eight-fold range), shape of survival curves for a given shear rate remained unchanged, while binding linear density varied proportionally to the amount of deposited ligand (see Fig. 3a,b). In this range, arrests were therefore considered as being the consequence of formation of single molecular bonds. We chose the second highest density (0.01 μg/ml) in this range for the following experiments.

Besides, we quantified the HLA A2 antigen deposited on these surfaces by immunofluorescence. The amount of HLA A2 antigen deposited at 0.01 μg/ml was of 1 molecule/μm^2^, which was consistent with single molecular association for evenly distributed ligand molecules on the chamber surface^24^,^33^. Initial off-rates were calculated as the initial slope of the bond survival curve (measured between bond detection to 0.5sec). Values ranged from 1.8s^−1^ for applied force of 38pN to 3.4s^−1^ for applied force of 228pN.

### Effect of microsphere height on association kinetics

**Figure 2:**
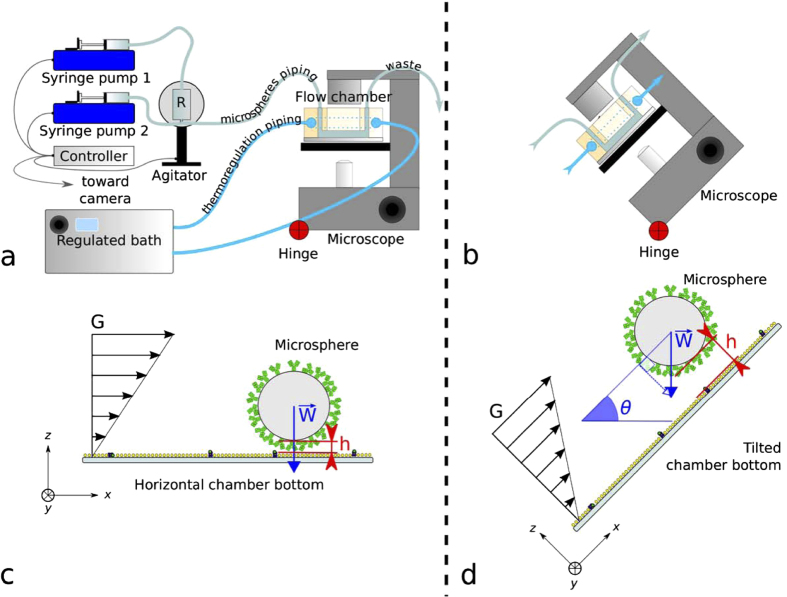
(**a**) new automated flow chamber set-up consisting of two syringe pumps, an agitator and a thermally regulated bath connected to a controller, and a flow chamber with inner thermal regulation piping mounted on a hinged microscope stage. A section of a single flow chamber is represented here for clarity (the actual device consists in eight independent chambers machined side-by-side in a single brass block) (**b**) tilted set-up. (**c**) principle view of the flow chamber. Distance (*h*) between microsphere surface and underneath chamber floor depends on its weight (*W*) and thermal energy. G is the shear rate. *h* is minimal with the chamber parallel to the horizontal plane. (**d**) principle view of a flow chamber tilted of angle *θ* reducing the component of weight directed toward the chamber’s wall and increasing *h*.

The probability of bond formation strongly depends on the distance between antibody and antigen, which determines the distribution of “encounter durations”. We systematically varied the average distance between the antibody-bearing microspheres and the antigen-bearing glass surface, and thus the experimental distribution of encounter durations. The time-averaged microsphere distance relative to an underlying horizontal surface depends on thermal motion, on microsphere weight and on surface forces that we measured under similar conditions in a previous work^24^. The microsphere lower surface was typically 30 nm above the underlying glass surface, comparable to the total length of the antigen-antibody bond that was *R* = 25 *nm*. We modified the microsphere-surface distance by changing the angle of the chamber’s bottom surface relatively to the horizontal plane: the component of gravity directing microspheres toward the chamber surface was reduced when the angle was increased from horizontal plane (see Fig. 2a,b). Average microsphere height thus increased with angle relative to horizontal plane (see Fig. 4a,b). Change in microsphere velocity due to the weight component parallel to the chamber surface was small relatively to the microsphere velocity range; modification of force applied on a ligand-receptor interaction was therefore also small, as was shown by numerical simulations of microsphere motion (see Fig. 4a). Thus, the distribution of encounter durations was experimentally varied as a function of chamber’s angle and of shear rate (see Fig. 4c for examples resulting of numerical simulations). Experiments were performed with the chamber set at 0°, 30°, 40°, 45°, 50°, 60°, 65°, 70°, 75° and 80° relative to the horizontal plane, and with 6 different shear rates for each tilt angle of the chamber. For each given angle a strong decrease of binding linear densities was seen when velocity increases (see Fig. 5a to j). A moderate increase of the off-rate (less than twofold, from 1.8s^−1^ to 3.4s^−1^) was also observed. Besides, for a given velocity, angle increase led to a strong diminution of binding linear densities (up to nine fold, see Figs 4d and Fig. 5a to i), while off-rates remained unchanged (data not shown).

We used numerical simulations to calculate the distributions of encounter durations for each experimental condition (see Fig. 4c). We compared two bond formation models by calculating for each experimental condition the binding linear densities predicted by our models according to distributions of encounter duration *t*_*e*_. One experimental condition consists of one angle of the set-up and one shear rate. We report in total n = 35 different experimental conditions represented each by one point in Fig. 5a to i and also Fig. 6a,b. The first bond model described bond formation with a classical on-rate *k*_*on* 2*D*_ (i.e., one free energy barrier leading to one free energy well), following Eq. 1,the 2D on-rate *k*_*on* 2*D*_ being the sole free parameter to fit experimental data. The second bond model defined bond formation as kinetically limited by slow diffusion through a rough part of energy landscape leading to a free energy well^23^. The rough energy landscape led to a minimal encounter duration and contained two free parameters, following Eq. 2.One adjustable parameter was the minimal encounter duration *t*_*on*_, the other adjustable parameter was the prefactor *f*_*E*_, with both values depending on bond geometrical parameters *φ*_max_ and Δ*R*_max_ (see Fig. 6) that define the diffusion volumes of both reactive species. We estimated the effect of Δ*R*_max_ and *φ*_max_ by calculating the values of binding kinetics obtained through each bond model with systematic variation of *φ*_max_ values (from 0.1rad to 1.5rad) and Δ*R*_max_ values (from 0.5 nm to 2 nm, see Fig. 7a,b). *f*_*E*_ parameter ranged from *f*_E_ = 0.2 for Δ*R*_max_ = 2 *nm* to *f*_E_ = 0.9 for Δ*R*_max_ = 0.5 *nm* (see Fig. 7c). While these parameters showed an effect on the quality of fit of the on-rate (*k*_*on* 2*D*_) model, they had very little effect on quality of fit by the rough landscape (*t*_*on*_) model (see Fig. 7d). To limit the number of free parameters, we set geometrical parameters at Δ*R*_max_ = 1 *nm* and *φ*_max_ = 0.5*rad* for further calculations and discussion as both values were reasonable, and as their effect on *t*_on_ was limited. Simulated binding linear densities were calculated for each experimental condition (i.e., angle of the set-up and shear rate) using the best adjustable parameters for the whole set of data, for both on-rate and rough energy landscape models. The on-rate (*k*_*on* 2*D*_) model best fitted data with *k*_*on* 2*D*_ = 22*s*^−1^ (correlation coefficient *r* = 0.870 ± 0.086), while the rough landscape (*t*_*on*_) model best fitted experimental data with prefactor *f*_*E*_ = 0.42 and minimal encounter duration *t*_*on*_ = 0.25 *ms* (correlation coefficient *r* = 0.959 ± 0.049), see Fig. 5a to j and also Fig. 6a,b. Overall, the rough landscape (*t*_*on*_) model fitted the experimental data better as was assessed by two different statistical tests: First, Fisher’s Z-test showed a significant difference between the two correlations, with p = 0.024. Second, by noting *e*_*i*_ the linear density of binding in experimental condition *i*, *s*_*i*_ was the corresponding simulated linear density of binding obtained from the global fit of the with one of the bond models, and *n* was the total number of experimental conditions, parameters of linear regression were written as follows:


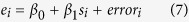


Expected values of the parameters were *β*_0_ = 0, *β*_1_ = 1. Parameters *β*_0_ and *β*_1_ may be estimated as


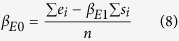


and as


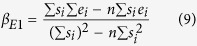


respectively for each bond models. The on-rate (*k*_*on* 2*D*_) model yielded values of *β*_*E*0_ = 0.00057, *β*_*E*1_ = 0.4287. The rough landscape (*t*_*on*_) model yielded better values of *β*_*E*0_ = 0.00021, *β*_*E*1_ = 0.8411. Finally, to rule out the possibility that two adjustable parameters in rough landscape (*t*_*on*_) could explain its better fitting of experimental results compared to the on-rate (*k*_*on* 2*D*_) model, we tested the fit of experimental data by the rough landscape (*t*_on_) model with several fixed values of prefactor *f*_*E*_ and a single free parameter (minimal encounter duration *t*_*on*_) (see Table 1): the rough landscape (*t*_*on*_) model with a single free parameter also fitted experimental data better than the on-rate model.

### Temperature and roughness measurement

We measured the kinetics of bond formation and rupture for temperatures 15 °C, 20 °C, 25 °C, 30 °C, 35 °C, 40 °C and 45 °C, during flow chamber experiments performed in single molecular bond condition, with a horizontal chamber and using the same 6 different shear rates. Bond formation kinetics increased with temperature (Fig. 8a), while bond rupture kinetics remained relatively unchanged (data not shown). We applied the rough energy landscape model for each experimental condition (i.e., temperature and shear rate) by calculating the binding linear densities predicted by our model according to distributions of encounter duration *t*_*e*_. We set the prefactor parameter at *f*_*E*_ = 0.15 in order to keep the bond intrinsic parameter *t*_*on*_ identical to the value obtained at similar temperature in the previous set of experiments. Correlation between experimental and simulated binding linear densities were calculated (Fig. 8a). The results again showed that the rough landscape (*t*_*on*_) model satisfactorily fitted experimental data, with correlation coefficient *r* = 0.934 ± 0.062. Linear regression parameters were calculated as shown for microsphere height variation assays, with *β*_*E*0_ = 0.00025, *β*_*E*1_ = 0.8080. The variation of *t*_*on*_(*T*) as a function of temperature *T* allowed us to estimate the roughness of the energy landscape. Conversely, we could not satisfactorily fit the Arrhenius law to the temperature dependence of *k*_*on* 2*D*_, further supporting the rough landscape (*t*_*on*_) model. The diffusion coefficient *D*(*T*) in a rough energy landscape varies as a function of temperature *T* following


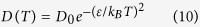


where *ε* is the roughness of the energy landscape^25^. We defined bond formation as the diffusive crossing of a rough section of the energy landscape, which depends on duration *l*^2^/*D*(*T*) where *l* is a parameter intrinsic to the bond with the dimension of a length^23^,^24^. Variation of *t*_*on*_ as a function of temperature can thus be written as


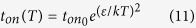


We fitted this function to experimental *t*_*on*_(T) measured at different temperatures to retrieve roughness *ε* (Fig. 8b). This gave approximatively *ε* = 7 × 10^−21^*Joule*, a value close to 2*kT* at 273K.

## Discussion

In this work, we measured association kinetics of an antibody and its antigen in two radically different conditions to allow comparison between 2D and 3D association. It was first measured in a classical 3D assay using surface plasmon resonance. In surface plasmon resonance assays, one molecular specie is tied to a surface, while the other diffuses freely in solution. Surface plasmon resonance retrieved here *k*_*on*_ = 1.4 ± 0.3 × 10^5^*M*^−1^*s*^−1^, in the usual range for antibody-antigen interactions. Second, association kinetics was measured in a 2D assay. In such assays, both molecular species are linked to surfaces. Several biophysical methods can measure 2D association kinetics at the single molecular level, including atomic force microscopy, optical tweezers, biomembrane force probe and the laminar flow chamber^23^,^24^,^31^,^34^. The latter method arguably provides the best way of controlling the duration of very brief encounters. Ligand bearing surface and receptor bearing surface are approached to put ligand-receptor in the vicinity and to allow interaction, then they are pulled apart for bond detection and lifetime measurement. The pulling phase implies a force exerted on the bond. This was done here with the laminar flow chamber, retrieving *k*_*on* 2*D*_ = 22*s*^−1^. We thus had the opportunity to directly compare on-rates measured in the two conditions. In principle, 2D and 3D quantities are related trough (see refs 31 and 35):


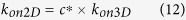


where c* represents the effective concentration of one ligand in the diffusion volume. Following our geometrical hypotheses (Fig. 6), the diffusion volume *V*_*φ* max_ is written:


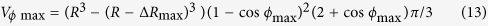


The effective concentration is written


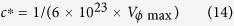


in moles per volume unit.

**Figure 3:**
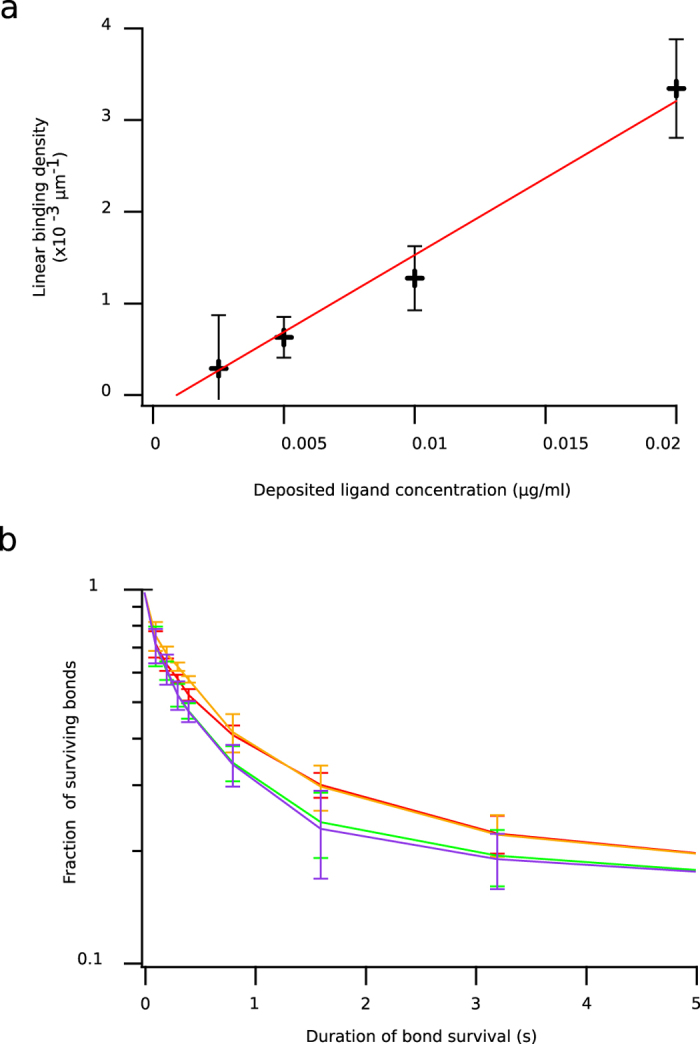
Proof of single molecular bond measurements: four HLA A2 densities (0.0025 μg/ml, 0.005 μg/ml; 0.01 μg/ml, 0.02 μg/ml) were used in the flow chamber, experiments were performed for 6 shear rates (or microsphere velocity). For one given shear rate, (here, 60s^−1^) binding linear densities (**a**) increased roughly linearly with the amount of ligand (red line is a linear fit of data), while the shapes of survival curves (with instantaneous slope equal to *k*_*off*_ at a given time point) did not change (**b**).

**Figure 4:**
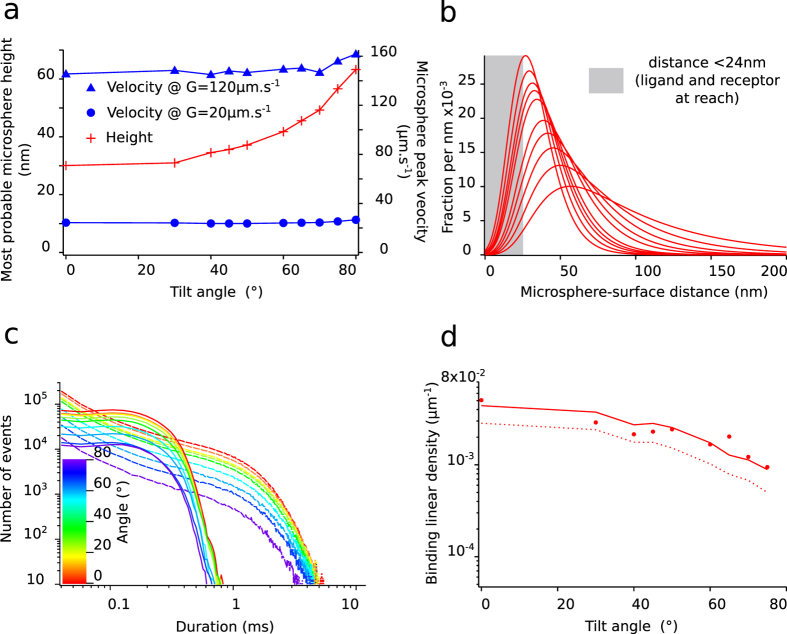
(**a**) example of results of the numerical simulation of microsphere movement in coordinates *x*, *y* and *Z*, following eqs 16, 17 and 18, showing the relationship between angle of the chamber relative to the horizontal plane and microsphere most probable height (red crosses) and microsphere velocity (blue circles for lowest shear, blue triangles for highest shear in our experiments). Velocity increase (and shear force increase) remained small compared to height increase. (**b**) Distribution of microspheres-surface distance for a given shear rate and angles ranging from 0 to 80° obtained by simulation. The area highlighted in grey represent the part of the distributions of height under 24 nm, putting ligand and receptor at reach. (**c**) examples of simulated distributions of encounter durations (as used in the simulations) for lowest shear (full lines) and highest shear (dotted lines) used in the experiments, with chamber tilt angles ranging from 0° (red) to 80°(purple). (**d**) example of experimental data showing binding linear density versus angle for a given shear rate (40s^−1^). Drop in binding linear density when angle relative to the horizontal plane increases is approximatively five-fold here.

**Figure 5:**
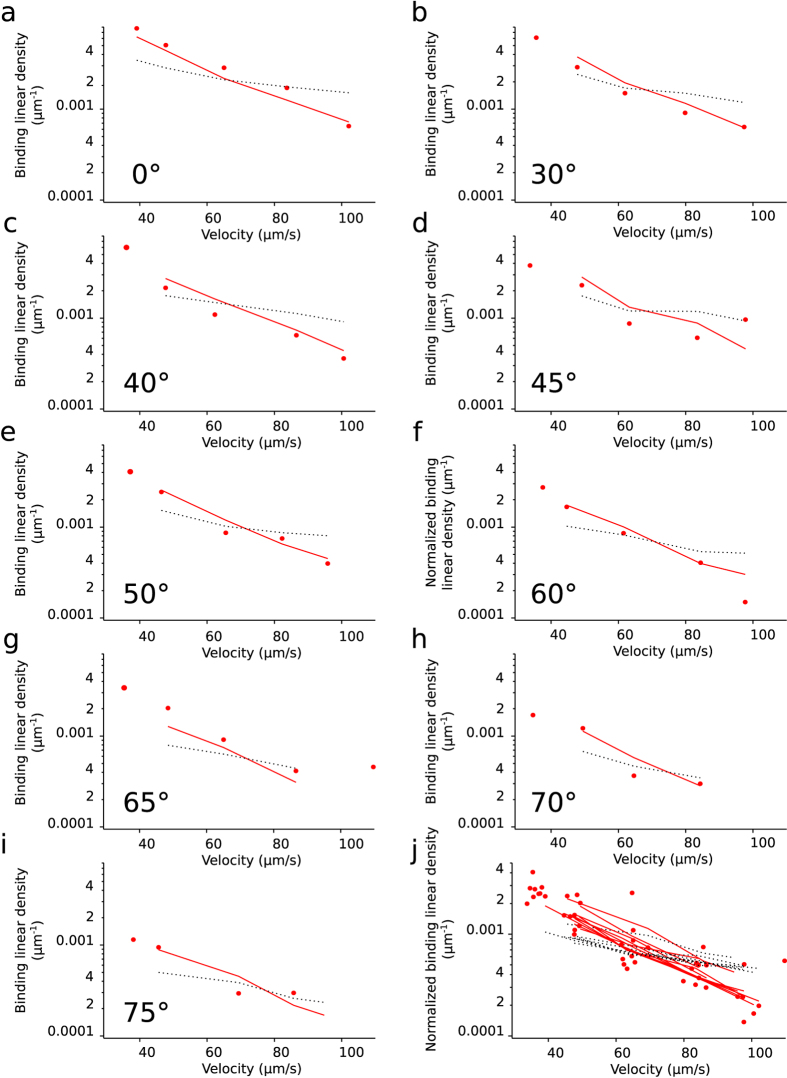
(**a–i**) Binding linear densities measured in single molecular bond conditions for various shear rates and various set-up angles: for a given angle, binding linear density is plotted versus average microsphere velocity, for angles ranging from 0 to 75° (80° is not plotted as adhesion was very low and only one single velocity condition had a significant number of adhesion events). For each individual graphic, results of a global fit of the data by either the t_on_ model (continuous red line) or the k_on_ model (dotted black line) for the corresponding condition in show the general better fitting of the t_on_ model. (**i**) Binding linear densities plotted versus average microsphere velocity after normalization. Normalization is done for a given condition by dividing the total number of adhesion events by the fraction of simulated microspheres trajectories during which the lower surface is below 24 nm (i.e., the fraction of time during which ligand and receptor are at reach, see Fig. 4B). Normalized fit of the data by t_on_ model is shown by a continuous red line for each condition, normalized fit of the data by the k_on_ model is shown by a dotted black line for each condition.

**Figure 6:**
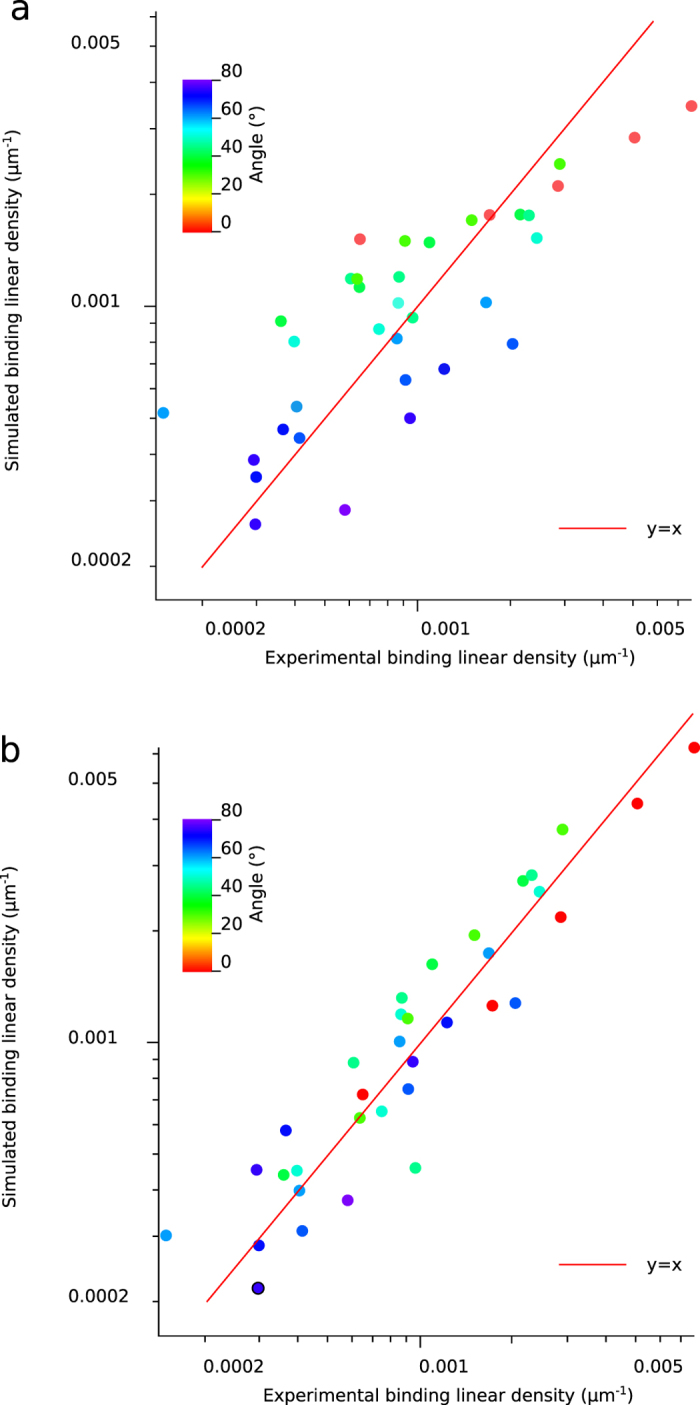
Comparison of association models for systematic changes in distributions of encounter durations obtained by changing shear rate and slope of the experimental set-up. Points of identical color were binding linear densities retrieved at identical angle but different shear rate. Simulated binding linear densities (left axis) are plotted against experimental binding linear densities (bottom axis): (**a**) on-rate model (correlation parameter *r* = 0.870 ± 0.086); (**b**) rough energy landscape model (correlation parameter *r* = 0.959 ± 0.049). Red line is a plot of equation *y* = *x* with *y* on left axis and *x* on bottom axis.

This yields *k*_*on* 3*D*_ = *V*_*φ*_ × 6 × 10^23^ × *k*_*on* 2*D*_ × 10^−15^ with *V*_*φ*_ in μm^3^ and *k*_*on* 2*D*_ in ms^−1^. Taking total bond length *R* = 24*nm*, Δ*R*_max_ = 1*nm* and *φ*_max_ = 0.5 *rad*, we obtained *k*_*on* 3*D*_ = 946M^−1^*s*^−1^. Strikingly the interaction displayed two orders of magnitude slower association rate measured in 2D than measured in 3D. While this result depends on the values of parameters Δ*R*_max_ and *φ*_max_, for a large range of these parameters, *k*_*on*3*D*_ remains largely inferior to the *k*_*on*_ value measured with surface plasmon resonance. Importantly, the laminar flow chamber measures association kinetics by counting individual binding events subjected to a disruptive force very shortly after binding: therefore, it measures interactions submitted to a certain amount of force, with a certain temporal resolution. As a consequence, single interactions with a sufficient strength and a sufficient lifetime may be selected, reflecting therefore a relatively deep part of the energy landscape. In contrast, surface plasmon resonance measures kinetics in the absence of force other than molecules thermal motion. The relevance of 3D measurements where short-lived or weak interactions account for association kinetics as well as longer interactions, and where no mechanical force is applied, is therefore questionable for 2D molecular interactions. We propose that the definition of 2D association kinetics should be function-oriented, that is, include a force resistance or lifetime parameter. In our experiments, the kinetics were measured with forces ranging from 38pN to 228pN, which match the order of magnitude of forces exerted by cells on receptors during the immune response^36^. The high difference between 2D and 3D association kinetics could be due to early breaking in 2D of short-lived bonds, not detected during flow chamber experiment but detected in surface plasmon resonance, and that could have evolved toward stronger bonds in the absence of force through the previously evidenced spontaneous maturation process^37^. Such an increase in initial off-rate could be due to direct force-facilitated rupture^2^, or to force-impaired reinforcement. Indeed, bond survival curves (see Fig. 3b) showed fast initial bond rupture, especially if compared with ruptures occurring after several seconds of lifetime.

We measured association kinetics in the laminar flow chamber while systematically varying the distribution of encounter durations between ligand and receptor, either through microsphere’s height variation or through shear rate variation. We found that the rough energy landscape model introduced earlier achieved significantly better correlation than the on-rate model with data. The rough energy landscape model uses two free parameters (minimal encounter duration *t*_*on*_ and prefactor *f*_*E*_), while the on-rate model uses one free parameter (on-rate *k*_*on*_). Comparison of on-rate model and minimal encounter duration model with a single free parameter and various pre-set *f*_*E*_ still showed better fit of experimental data by the minimal encounter time model (see Table 1). We interpreted the minimal encounter duration *t*_*on*_ as a direct consequence of slow diffusion through the rough part of the landscape and it was the main value describing bond formation. We interpreted the prefactor *f*_*E*_ (that is rather close to one) as a phenomenological parameter that represent the proportion of functional ligand and receptor on the surfaces, with losses due to protein denaturation or incorrect orientation. In an on-rate model, this aspect would be lost, as any loss of functional ligand or receptor would appear as a lowered on-rate.

As a major function of proteins is to bind other proteins or molecules, association kinetics are of primary importance, and the classical on-rate model has proved extremely useful. Several methods, as described earlier, are able to measure association kinetics at the single molecular level (atomic force microscopy, optical tweezers and biomembrane force probe). Yet, all of them present relatively large encounter durations (50 ms in a study using biomembrane force probe^34^; 60 ms in a study employing AFM^31^). Other methods operating in 2D conditions but not at the single molecular level exist, such as the “thermal fluctuation assay”^38^ or interferometry of giant liposomes^39^. In these methods, neither a disruptive force nor encounter durations are controlled^40^,^41^. In addition, several theoretical works have quantified association rates between moving surface-bound molecules^21^,^29^,^30^,^40^,^42^, including the effect of a compressive force^41^. In this body of work, intrinsic bond association kinetics was described as an on-rate. Yet, the on-rate model arose experimentally from solution chemistry, where encounter durations are governed solely by thermal agitation and are not otherwise experimentally adjustable, and where, conceptually, single activation energy peaks are often suitable to describe energy landscapes. In a laminar flow chamber, the effect of change in the distribution of encounter durations on molecular interactions can be measured. Short encounter durations are generated, distributed from 0.1 to 10 ms. The data obtained here challenge the on-rate model, and favor a minimum encounter duration model. A complex landscape with successive energy wells may also account for this set of data, but the high number of free parameters in such a model renders its validation difficult^23^. Roughness appears as a robust means to describe this complex part of the energy landscape that leads to binding. The rough part of the energy landscape might contain the short-lived or weak bound states envisioned earlier, that would not be detected by the laminar flow chamber but by surface plasmon resonance. Indeed, the 2kT roughness value is of the order of magnitude of the binding energy of some surface-bound molecular interactions^43^. It is also comparable with the order of magnitude of roughness measured during dissociation of biotin-streptavidin bond (4.5kT or 8kT ± 1kT)^40^,^44^ or during folding and unfolding of N terminal domain of phosphoglycerate kinase (4kT to 5kT)^45^. Roughness could results from diffusive displacement of peptidic chains before the actual free energy gain may take place^46^,^47^. Such conformational change suits the model of antigen-antibody binding as the sum of numerous weak interactions between amino acids residues after conformational adjustment^48^.

The 2D quantification of antibody-antigen or BCR-antigen kinetics under force is fully relevant to the physiology of the immune system, as molecular interactions taking place at surface-surface interfaces are indeed common. A most important case is the interaction between a BCR and its ligand in a lymph node, during the initial detection of its ligand by a B cell that may trigger its activation. B lymphocytes seem to detect mainly the affinity of BCR-antigen interaction^49^ at a very early stage, through poorly understood mechanisms. These may include the facts that B cell pulls on the antigen^10^ thus strongly reducing the lifetime of BCR-antigen bonds, and that BCR forms very early oligomers then microclusters^50^ at interaction sites with the antigen, without the need for multivalent antigen^10^,^51^. In order to decipher how the B lymphocyte may sense affinity through oligomer formation, we suggest that strong signaling differences could arise from bonds whose association kinetics are governed by minimal encounter durations rather than by on-rate. Oligomerization depends on association kinetics of forming interactions, during a time limited by dissociation kinetics of already established interactions. Following an on-rate model, the number of newly formed interactions would be linearly dependent on lifetime of formed interactions. Strikingly, a minimal encounter duration would act as a threshold: only interactions with lifetime beyond the minimal encounter duration would ensure oligomerization, and thus signal propagation. This could be a way to discretize cell response to ligand-receptor binding properties, on a short timescale as observed during B cell responses^49^. In T-cell, in a recent work, force generation and transmission through TCR was linked to the activation potency of ligands prior to cell activation, showing supramolecular complex formation implying LCK while under tension^36^. More generally, the modulation of t_on_ might allow cells to analyze surrounding surfaces likely to expose diverse ligands with brief touches of controlled duration.

## Conclusion

The laminar flow chamber allows to measure 2D association kinetics of biomolecules. 2D and 3D association kinetics of an antibody-antigen bond are strongly different, suggesting that a relevant quantification of 2D binding should include a reference to force. Here, association kinetics are measured in a force range relevant to cell biology. The laminar flow chamber offers also a unique control on the distribution of short-lived encounters durations that challenges the classical on-rate model. A rough energy landscape model appears more suitable than a single activation energy landscape to describe antibody-antigen or BCR-antigen binding, with roughness evaluated at 2*k*_*B*_*T*. Additionally, we describe an enhanced laminar flow chamber set-up for time-efficient and enriched quantification of 2D binding kinetics.

## Materials and Methods

### The automated flow chamber apparatus

We built a new laminar flow chamber set-up with automated agitation and injection of microspheres, automated camera control and automated change of shear rate; additionally the microscope stage could be manually tilted, and sample temperature could be chosen. The experimental set-up is as follows (more details are given in Supplementary Informations section, including Supplementary Fig. 2). Eight independent chambers (8 × 2 × 0.15 *mm*^3^) were machined side-by-side in a single brass block roughly the size of a glass slide (75 × 25 *mm*^2^), forming a multi-chamber device. Each chamber had a clear PMMA window for sample illumination. A single glass slide formed the bottom of the eight chambers, which were separated by their individual gasket. An inner piping was machined at the periphery for circulation of temperature regulating fluid. The complete set-up (see Fig. 2a) consisted in the multi-chamber device set on an inverted microscope (Leica, Germany) equipped with a video camera (Sony, France) and a ×20 objective lens. A controller (based on a Mega2560 microcontroller, Arduino, Italy) actuated an agitating device holding a reservoir for microsphere suspension connected to one chamber entry, a first syringe pump connected to the microsphere reservoir, a second syringe pump connected to the chamber entry, and controlled microscope illumination. During a typical operating cycle, microsphere suspension was agitated, then injected in the chamber by the first syringe pump. The second syringe pump then established the shear flow at a chosen shear rate, while the camera (IDS, Germany) recorded microsphere displacement (see Fig. 2c) at 50 images per second. Movies were compressed on-the-fly by the IDS U-Eye software using its native M-JPEG codec. The automaton repeated such cycles with a new shear rate until all chosen shear conditions were recorded. Piping was then manually connected to the next chamber. Microsphere height could be controlled by varying the component of gravity directed toward chamber’s bottom (Fig. 2b,d). For this purpose, the microscope and flow chamber were tilted relatively to the horizontal plane, the microscope being bolted to a plate hinged to the bench and held at a chosen angle. Temperature was controlled through circulation of water from a thermally regulated bath in the dedicated piping of the flow chamber device.

### Microsphere and surfaces preparation

The functionalized surfaces used in the flow chamber were prepared as follows: 75 × 25 mm^2^ glass slides (VWR, France) were rinsed twice in ethanol then in water. The glass slides were cleaned in a “piranha” solution, a heated mix of 70% H_2_SO_4_ (Fisher Bioblock, France) and 30% H_2_O_2_ (50% in water, Sigma-Aldrich, France), for ten minutes, rinsed and stored in deionized water. The glass slides were coated with a poly-L-lysine solution (150000–300000Da, Sigma-Aldrich, France) in 0.02 M phosphate buffer, pH 7.4, 100 μg/ml for 30 minutes, rinsed in phosphate buffered saline (PBS), then incubated with a glutaraldehyde solution (2.5% in 0.1 M borate buffer, pH 9.5, Sigma-Aldrich, France) for 10 minutes, and rinsed in PBS. Glass slides were then incubated with a saturating solution of biotinylated bovine serum albumin (BSA) (100 μg/ml, Sigma-Aldrich, France) in PBS, for 30 minutes, then rinsed with PBS. Glass slides were incubated for 30 minutes in a blocking solution of glycine (0.2 M) and BSA (1 mg/ml) in PBS, rinsed in PBS, then incubated in a saturating streptavidin solution (10 μg/ml in PBS, Sigma-Aldrich, France) for 30 minutes, then rinsed with PBS. One glass slide was mounted in the multi-chamber device, with each well afterwards independently incubated with biotinylated Human Leukocyte Antigen A2 (HLA A2, a Major Histocompatibility Complex class II molecule with antigenic peptide) at a given concentration. HLA A2 molecules were expressed in *E. coli* from amino acid 1 to amino acid 278, corresponding to the entire extracellular domain plus 4 amino acids; a biotinylation sequence of 15 amino acids for BirA enzyme was added at the C-terminal end, with biotin linked to the tenth amino acid of this sequence. Functionalized microspheres were prepared as follows: Dynabeads M450 Tosylactivated microspheres (diameter: 4.5 μm, Invitrogen, France) were coated with a monoclonal mouse anti-human HLA A, B, C antibody (MCA485G, Serotec, France), according to the manufacturer’s protocol. Briefly, microspheres were rinsed in 0.1 M pH 9 borate buffer, incubated 24 hours at 37 °C in an antibody solution in 0.1 M pH 9 borate buffer, then rinsed in PBS, and incubated in blocking solution of TRIS 0.1M and BSA 0.1% for 4H at 37 °C. Between experiments, microspheres were stored in this solution at 4 °C with 0.01% sodium azide added.

### Trajectories analysis and arrest statistics

Statistics of bond formation was determined by counting the number of microspheres arrests and the total distance travelled by microspheres after sedimentation, as previously described^5^. Statistics of bond rupture was determined by measuring the durations of microspheres arrests (Java plug-ins incorporated in ImageJ (NIH, USA) were written for both purposes). Briefly, a microsphere was considered to be arrested if its position did not change by more than *dx* = 0.5*μm* during *τ* = 0.2*s*, and if its velocity before the arrest was within the velocity range of microspheres moving in the shear flow after sedimentation. This range was defined from the histogram of microspheres velocities, as the velocity interval bordering the peak of microsphere velocities that is due to sedimented microspheres. The interval was set at two times the width of the peak at half its maximum height. An arrest was considered to continue as long as the arrest criterion was satisfied, which yielded an apparent duration *d*_*app*_. The true arrest duration *d*_*true*_ was obtained with the correction


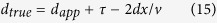


where *v* was the mean velocity of microspheres moving in the shear after sedimentation^52^. The binding linear density under a given condition (*i.e.*, a given shear rate, a given ligand surface density, a given temperature and a given set-up angle relative to the horizontal plane) was defined as the number of arrests divided by the total distance travelled by the microspheres after sedimentation. The binding linear density of specific association was calculated by subtracting from the binding linear density measured with assay surfaces the binding linear density obtained with control surfaces (without HLA A2 molecules). Statistics of bond rupture under a given condition (*i.e.*, a given shear rate, a given ligand surface density, a given temperature and a given set-up angle relative to the horizontal plane) were described by building survival curves of the bonds, obtained by counting the fraction *S* of arrests exceeding the duration *t* versus *t*. Standard deviation *SD* was calculated as the experimental SD between individual experiments preformed in identical conditions. The specific binding linear densities for the reference condition used here (25 °C, horizontal chamber, single molecular bonds observed at 0.01 μg/ml, and 6 different shear rates ranging from 20s^−1^ to 120s^−1^) were in average more than twelve time higher than the non-specific binding linear densities under the same conditions. Value of this specific over non-specific binding linear densities ratio ranged from 5 to 39 for all shear rates ranging from 20s^−1^ to 100s^−1^ in these conditions, allowing proper measurement of antigen-antibody bond survival and assessment of single molecular bond measurement. This ratio was lower in conditions strongly reducing antibody-antigen interactions (highest shear rates and steep angle of the chamber relatively to horizontal plane). This was not considered a concern as in this work only binding linear densities were considered in such conditions and not bond durations.

### Surface plasmon resonance measurements

Surface plasmon resonance experiments were performed using a BIACore T200 (General Electric Healthcare, USA). Surfaces were coated with the biotinylated HLA A2 (0.84 μg/ml perfused for 60s at 30 μl/min) and passivated by BSA (0.01% in PBS infused for 60s at 30 μl/min), while the mouse anti-human HLA A, B, C antibody was used in soluble form (1.88 nM, 3.75 nM, 7.5 nM, 15 nM, 30 nM and 60 nM). Kinetics of association and dissociation were followed through measurement of the surface plasmon resonance signal in single cycle kinetic mode, without regeneration (measurement durations were 480s for association and 600s for dissociation); affinity was measured at equilibrium. Association rate and affinities were obtained by standard fitting of the binding curve using the BIACore analysis software (see Supplementary Fig. 1). Experiments were repeated twice under each condition with consistent results.

### Immunofluorescence for HLA A2 quantification

Principle is to tag HLA A2 molecules on typical flow chamber surfaces with a fluorescent antibody, to measure the fluorescence signal per area unit, then to compare it with the fluorescence signal per area unit of known amounts of the same fluorescent antibody in solution forming thin layer between a glass slide and a coverslide. Test glass slides were prepared as for flow chamber experiment, mounted in the laminar flow chamber and coated with 1 μg/ml, 0.1 μg/ml or 0.01 μg/ml of biotinylated HLA A2 solution, then rinsed twice with PBS, then incubated with 5 μg/ml fluorescent anti-HLA antibody solution (Serotec, France) in the chamber for 20 minutes, then rinsed twice with PBS. The calibration was performed with solutions of fluorescent anti HLA antibody (Serotec, France) of 50 μg/ml, 5 μg/ml, 0.5 μg/ml, 0.05 μg/ml and 0.005 μg/ml in 0.1% BSA in PBS, with 5 μl of each solution deposited between a glass slide and a 22 × 22 *mm*^2^ coverslide and sealed with nail varnish. Fluorescence signal was measured by a camera (Andor, France) on an Axiovert 200 microscope with a ×20 objective with a numerical aperture of 0.8 (Zeiss, Germany); a calibration curve was drawn from signal from antibody solutions then compared to signal from surfaces prepared as for flow chamber experiments for quantification.

### Numerical simulations

Numerical simulations were used to assess the microsphere motion and distribution of molecular encounter durations as a function of experimental conditions (shear rate, set-up tilt angle, temperature), they combine dynamics of a microsphere in laminar flow with a calculation of the diffusion volumes of antibody and ligand reactive sites. A molecular encounter was defined to begin and last as long as the diffusion volume of a receptor (or antibody molecule) intersects the diffusion volume of a ligand (or HLA A2) molecule.

Brownian dynamics of a bead in a laminar flow near a wall was calculated as follows. A bead of radius *α* is convected by a laminar flow of shear rate *G* at a distance *z*from a wall. *x* and *y* are the coordinates in the plane of the chamber floor respectively parallel and perpendicular to the flow. Equations for constructing the trajectory of the bead are modified from^24^ to account for the tilt of the flow chamber by an angle θ from the horizontal plane:


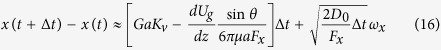



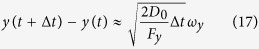



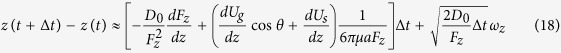


*D*_0_ = *k*_*B*_*T*/6*πμa* is the bulk diffusion coefficient in absence of wall. *μ* is the medium viscosity, which is taken to be the one of water. Δ*t* is the time step of the simulation. *ω*_*x*_, *ω*_*y*_, *ω*_*z*_ are random numbers, with a Gaussian distribution of width equal to 1, and the functions *F*_*x*_, *F*_*y*_, *F*_*z*_ and *K*_*v*_ account for the hydrodynamics friction next to the wall. They are evaluated at *z*(*t*), following


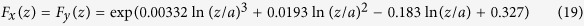



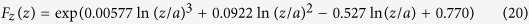


and


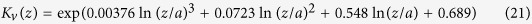


using a cubic approximation of numerical results provided by Goldman *et al.*^53^,^54^ as done in our previous works^24^,^32^. The microsphere potential is the sum of gravity potential *U*_*g*_(*z*) and interaction with the surface potential *U*_s_(*z*) following


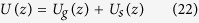


The microsphere-surface potential *U*_*θ*=0_(*z*) has been measured previously, in a horizontal configuration (corresponding to θ = 0)^24^. Briefly, the method is based on the measurement of the bead-surface distance by Reflection Interference Contrast Microscopy^52^. Microbeads imaged in RICM appears as Newton’s rings, the radius of which is related to the bead-surface distance, through a calibration established previously^52^. The statistical distribution of bead-surface distance *z* is obtained from the time-sequence recording of several beads. The histogram of the *z* distribution *φ*_*θ*=0_(*z*) is used to deduce the bead-surface potential *U*_θ=0_(*z*) in the form


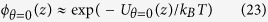


The force of interaction *dU*/*dz* is derived from the measured potential *U*(*z*) and approximated with the formula


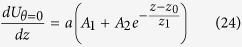


with a the microsphere radius, allowing to retrieve parameters A_1_ = −0.1 μN/m, A_2_ = 0.5 μN/m, z_1_ = 17 nm, z_0_ = 0 nm. For non-zero angle θ, force of interaction *dU*/*dz* is written (see ref. 24)


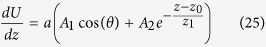


The initial position of the beads is set in order to follow the bead height distribution


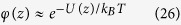


using a rejection method^24^. The influence of microsphere rotation is taken into account as follows^24^: shear-induced rotation of the microsphere is not modelized, but its effect is calculated as a 0.43-fold reduction of relative surface velocity, effect of rotational diffusion can be largely neglected in our conditions, as demonstrated earlier. Indeed, the rotational diffusion time becomes less than the convection time only for the lowest shear rates and z~L. The same set of equations is used to account for the effect of temperature on brownian motion, with tilt angle *θ* = 0 in this case, and explicit dependence of viscosity on temperature^55^.

**Figure 7:**
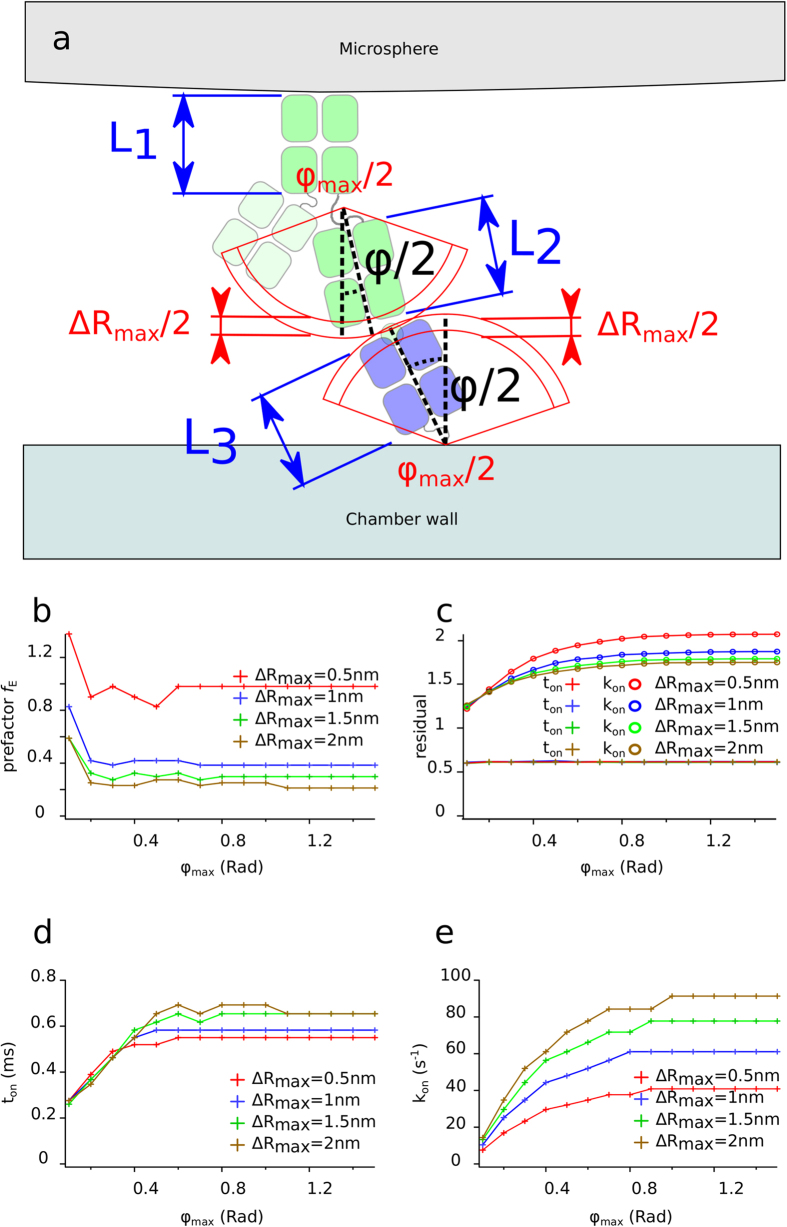
Molecular geometry used to simulate encounter durations. (**a**) Antibodies were linked covalently to a microsphere’s surface (grey, top), and were hinged between Fc fragment (of length L1 = 8 nm) and Fab fragment (of length L2 = 8 nm) through a 6 amino acids chain; HLA A2 (of length L3 = 8 nm) were linked to a streptavidin surface through a BirA sequence-linked biotin. We assumed that both binding sites were able to diffuse in shell-shaped volumes described by their thickness ΔL2 and ΔL3 both equal to ΔR_max_/2 (in red) and respectively angle *φ*_1_ and angle *φ*_2_ (both in red with maximum equal to *ϕ*_max_/2). *Effect of systematic variation of bond geometry parameters:* Effect of bond length play Δ*R* = Δ*L*2 + Δ*L*3 and of maximum rotation angle *φ*_max_ = *φ*_1_ + *φ*_2_ on calculated *k*_*on*_ and *t*_*on*_ obtained by fitting all experimental data with each bond model: (**b**) prefactor *f*_*E*_ in the *t*_*on*_ model plotted versus *φ*_max_ for ΔR_max_ varying from 0.5 nm to 2 nm; (**c**) effect of systematic variation of ΔR_max_ and *φ*_max_ on residuals from fits of all experimental data with each bond model. A residual *res* was defined as 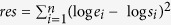 where *e*_*i*_ was the binding linear density in experimental condition *i*, *s*_*i*_ was the corresponding simulated binding linear density obtained from the global fit of the data with one of the bond models, and *n* = 35 was the total number of experimental conditions; (**d**) *t*_*on*_ plotted versus *φ*_max_ for ΔR_max_ varying from 0.5 nm to 2 nm; (**e**) *k*_*on* 2*D*_ plotted versus *φ*_max_ for ΔR_max_ varying from 0.5 nm to 2 nm. On the whole, *t*_*on*_ model was less dependent on bond geometry parameters than *k*_*on* 2*D*_ model, while residual of *t*_*on*_ model was systematically smaller.

**Figure 8:**
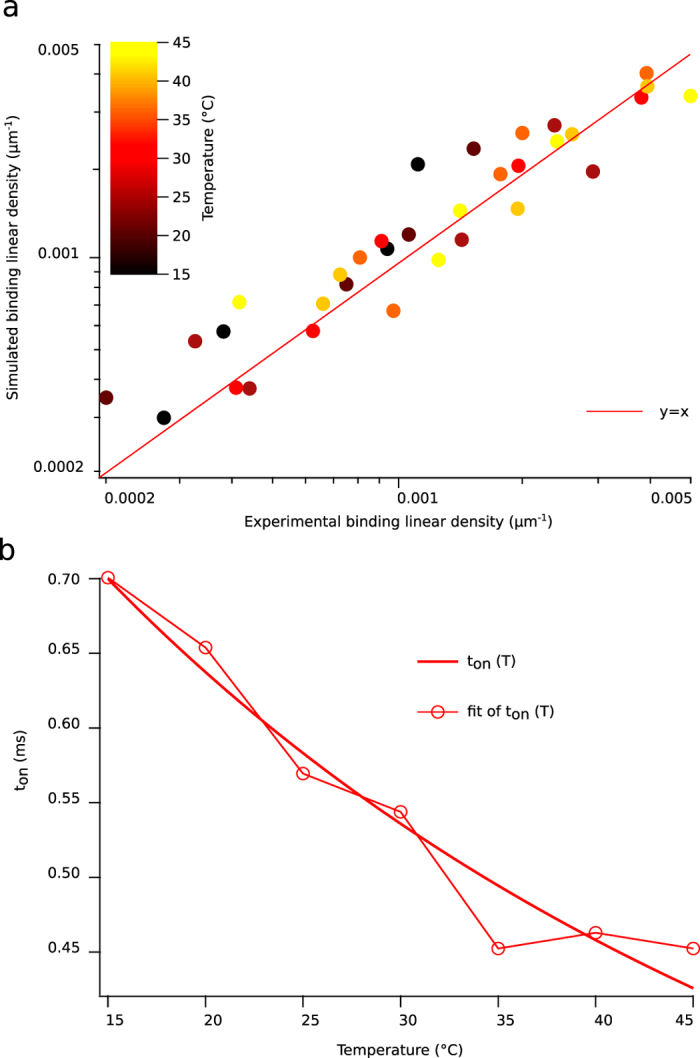
Results of systematic changes in temperature and shear rate on binding linear density. Points of similar color were binding linear densities retrieved at identical temperature but different shear rate; (**a**) data of simulated binding linear densities (left axis) plotted against experimental binding linear densities (bottom axis) with *t*_*on*_ model, showing good correlation between rough energy landscape model and experimental data. Correlation parameter is *r* = 0.934 ± 0.062. Red line is a plot of equation *y* = *x* with *y*on left axis and *x* on bottom axis; (**b**) experimental plot of *t*_*on*_(*T*) versus temperature *T* and fit of these data with the function 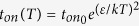
11, retrieving ε ≈ 2*k*_*B*_*T* at 25 °C.

Calculation of diffusion volumes of reactive sites is as follows. Antigen-binding site is at the extremity of the Fab fragment of the antibody; Fc fragment (of length *L*_1_ = 8 nm) and Fab fragment (of length *L*_2_ = 8 nm) are hinged through a 6 amino acids chain. Recognized epitope is on the distal α_1_ domain of the HLA A2 molecule (of length *L*_3_ = 8 nm), while the C-terminal end of the HLA α_3_ domain is linked to the biotin by a 14 amino acids chain. We assume that in both molecules chains non included in immunoglobulin domains give degrees of rotational freedom and some length variability considered as follows: the anchoring points on each surface are separated by a distance equal to (*L*2 + *L*3) ± Δ*R*_max_/2; the respective azimuthal angles *φ*_1_ and *φ*_2_, defined as the angles between the segments linking the anchoring points and the vertical, are *φ*_1_ < *φ*_max_/2 and *φ*_2_ < *φ*_max_/2 (for simplicity we lumped these conditions in *φ*_1_ + *φ*_2_ < *φ*_max_). In summary, both binding sites are able to diffuse rapidly in shell-shaped volumes described by their thicknesses Δ*L*2 and Δ*L*3 respectively with Δ*L*2 + Δ*L*3 = Δ*R*_max_ and by their angles *φ*_1_ and *φ*_2_ respectively (see Fig. 7).

The duration of molecular encounter between a ligand (on chamber floor) and a receptor (on bead surface) is estimated with several physical assumptions (see also^24^): (i) the density of ligand is low and the density of receptors is high; (ii) reactive sites describe shell-shaped diffusion volumes described above (see Fig. 7); (iii) the encounter starts as soon as and hold as long as geometrical conditions defining the intersection of diffusion volumes are fulfilled. These geometrical conditions are defined by the distance and angle between the reactive sites (see Fig. 7). The numerical simulation records all the positions fulfilling the above rules, using a time step Δ*t* = 0.01 *ms*. As hypothesized in (iii), each encounter duration *t*_*e*_ corresponds to the number of successive time steps where the geometrical conditions are continuously satisfied.

## Additional Information

**How to cite this article**: Limozin, L. *et al.* A Rough Energy Landscape to Describe Surface-Linked Antibody and Antigen Bond Formation. *Sci. Rep.* **6**, 35193; doi: 10.1038/srep35193 (2016).

## Supplementary Material

Supplementary Information

## Figures and Tables

**Figure 1:**
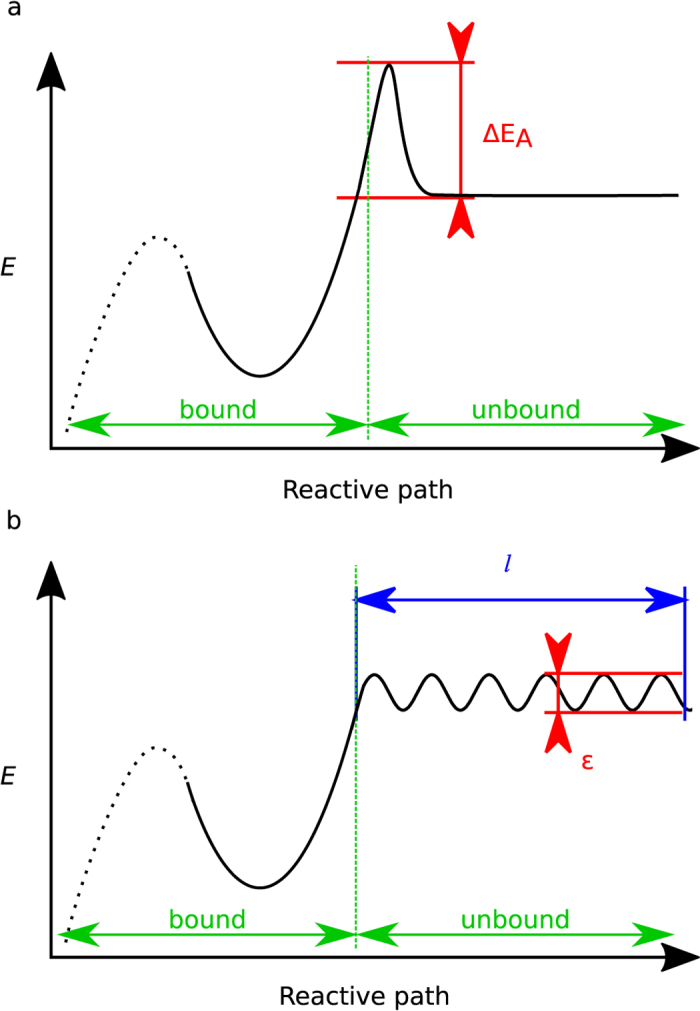
Two alternative energy landscapes for bond formation. (**a**) classical energy landscape formed by a free energy peak Δ*E*_*A*_ followed by a free energy well (only the first well of several possible is shown, with further parts of energy landscape suggested by dotted line). Probability of crossing Δ*E*_*A*_ as a function of encounter duration *t*_*e*_ is given by *P*(*t*_*e*_) = 1−exp(−*k*_on_ × *t*_*e*_)^1^. (**b**) rough energy landscape with numerous small peaks resulting in first part of roughness *ε* and length *l* followed by a free energy well (again, only the first well of several possible is shown, and further parts of energy landscape are suggested by dotted line). Probability of crossing the rough part of the energy landscape as a function of encounter duration *t*_*e*_ is given by 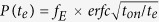
2.

**Table 1:** Residuals of fit of experimental data by minimal encounter duration model with *t*_*on*_ as the sole free parameter and several fixed values of prefactor *f*_*E*_.

Minimal encounter duration model	Fit residual
*f*_*E*_ = 0.042	1.46
*f*_*E*_ = 0.1	0.97
*f*_*E*_ = 0.42	0.63
*f*_*E*_ = 1	0.79
On-rate model	1.79
