# Supplementary material for: A Rough Energy Landscape to Describe Surface-Linked Antibody and Antigen Bond Formation
Source: Sci Rep. 2016 Oct 12;6:35193. doi: 10.1038/srep35193 (PMC5059681; doi:10.1038/srep35193)
Supplement: Supplementary Information [file srep35193-s1.pdf]

Supplementary information to

A Rough Energy Landscape to Describe Surface-Linked Antibody and Antigen Bond Formation.

L.Limozin, P.Bongrand, and P.Robert

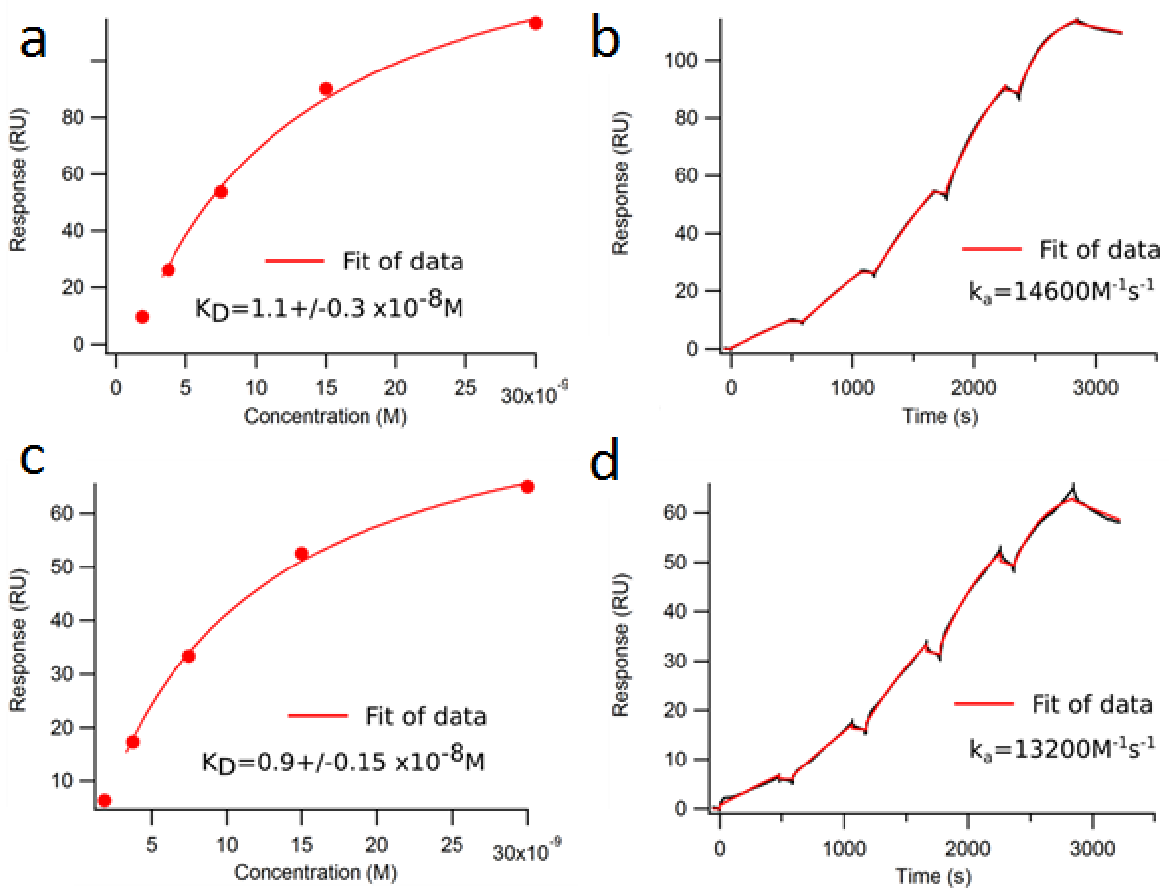

Supplementary Figure 1: Surface plasmon resonance experiments performed using a BIACore T200 (General Electric Healthcare, USA). Surfaces were coated with the biotinylated pMHC and passivated by BSA while the mouse anti-human HLA A,B,C antibody was used in soluble form. **a**, surface plasmon resonance affinity measurement at equilibrium for first experiment**. b,** kinetics of association measured in first experiment through measurement of surface plasmon resonance signal in single cycle kinetic mode, without regeneration. **c**, surface plasmon resonance affinity measurement at equilibrium for second experiment**. d,** kinetics of association measured in second experiment through measurement of surface plasmon resonance signal in single cycle kinetic mode, without regeneration.

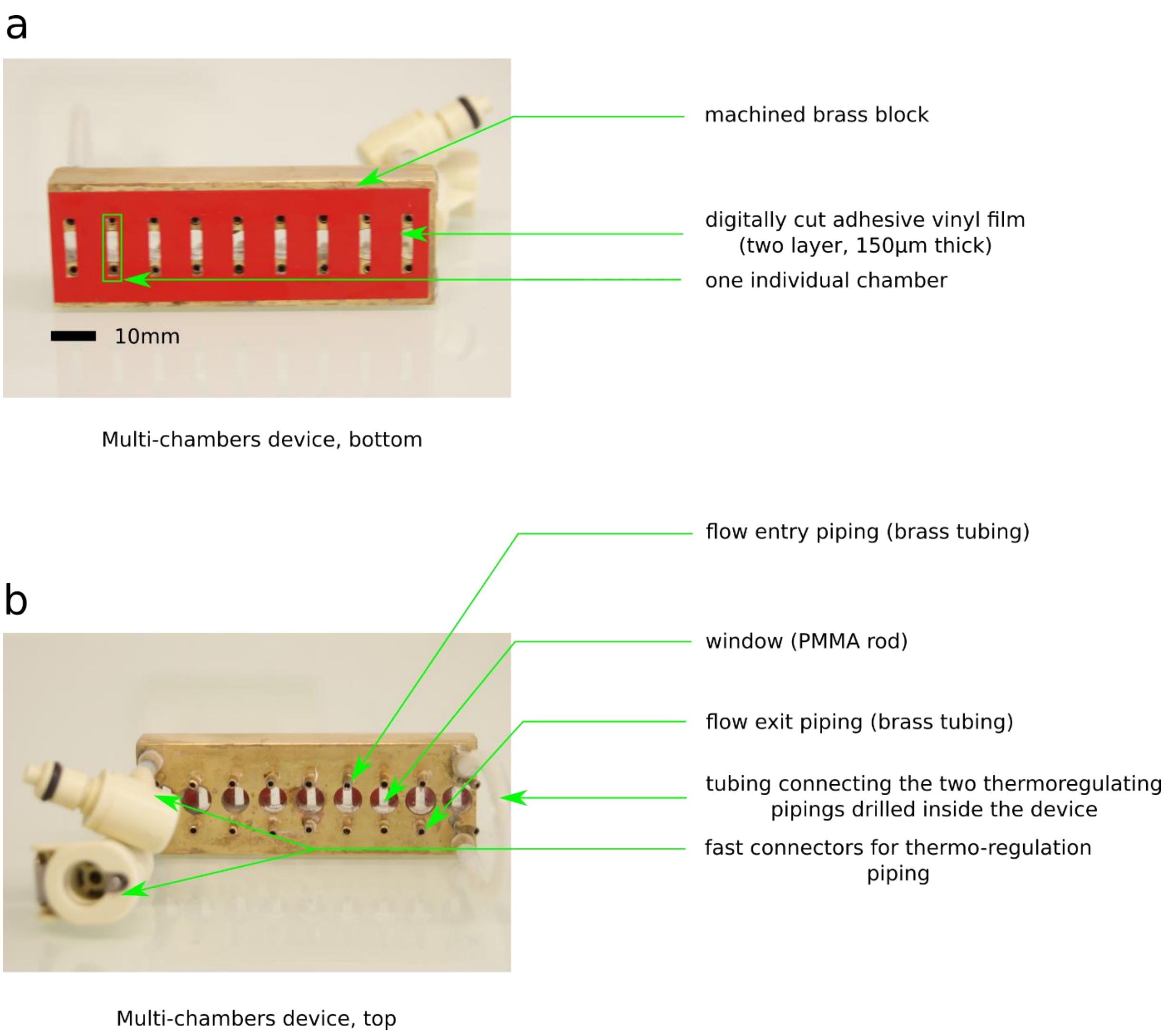

Supplementary figure 2: **a**, bottom of the device showing the nine individual chambers. **b**, top of the device showing the connections for laminar flow and microsphere injection, and also for thermal regulation.

Supplementary information on the automated laminar flow chamber set-up:

The laminar flow chamber device: the laminar flow chamber device is a block of brass, 75x25x12 mm^3 with several machined features. First, nine vertical cylindrical holes (diameter 6mm, one per individual chamber) are filled with PMMA rod to form illumination windows. Second, one entry hole on one side of each PMMA window and one exit hole on the other side are drilled, then brass tubing is glued to form connections for flow chamber PDMS piping. Third, two horizontal holes (diameter 4mm, length 75mm) are drilled on the outer side of the entry and exit holes, along the whole length of the device. Both extremities of both holes are filled with machined brass rod that are tin-welded to the brass block. Vertical holes (diameter 4mm) are then drilled at each extremity, with brass piping glued for connection to temperature control PDMS piping. An automated vinyl cutter (Graphtec, Japan) cuts nine rectangular holes (10x2 mm^2) in a 75x25mm^2 rectangle of 75µm thick self-adhesive vinyl film. Two such layers are

glued to the bottom of the machined brass block, forming nine individual $10x2x0.15mm^3$ chambers.

The temperature control system: The heating resistor of a bath heater (Thermo-Fisher, France) and the compressor, radiator, and whole coolant piping of an ice-cube machine are set in a plastic box (RS Components, France) with the heating resistor and cube-forming part of the ice-cube machine both set in an insulated plastic container of approximatively 3 liter volume, filled with water. PDMS piping is connected to the insulated container, then to a low-voltage water pump (RS Components, France), then to the flow chamber thermoregulation inner piping, then back to the water container. A microcontroller (Arduino Uno, Arduino, Italy) receives signal from a thermistor and controls both the heating resistor and the cooling compressor through relays. The microcontroller is programmed to regulate the container water temperature according to one of 10 pre-programmed settings, chosen through a rotating selector, and to pump the water at will in the chamber through temperature control PDMS piping.

The agitation device: A rectangular piece is machined in polyacetal (POM-C); a standard hobby servomotor (Hitec, Japan) is bolted to it with rubber bushing allowing some play, while its base is bolted to the microscope stage. A clamp able to hold the reservoir is machined in POM-C and bolted to the rotor of the servomotor. A vibrating electrical DC engine, identical to what is found in mobile phones, is fitted to the clamp. The reservoir itself is machined in a PMMA cylinder (diameter 20mm, length 50mm).

The laminar flow chamber automaton: The control device is based on a microcontroller (Arduino Mega, Arduino, Italy) that controls directly the servomotor and vibrating motor of the agitation device, the microscope illumination through a relay, and controls the syringe pumps stepper motors through dedicated stepper motors control boards (model A4983, Pololu, USA) with micro-step resolution. Both syringe pumps are made from the frame and lead-screw of former single-speed syringe pumps (Razel, USA) and equipped with stepper motors (Crouzet, France).

Experimental sequence and movies post-processing: Prior to data acquisition, microsphere reservoir is filled with microsphere suspension, injection syringe, flow syringe, piping and one chosen chamber are filled with PBS with 0.2% bovine serum albumin. Thermoregulation bath is connected to the chamber and its temperature selected. The operator launches a movie acquisition by the camera software, then the operator turns the automaton on. What follows is fully automated: microsphere reservoir is agitated by rotation and vibration, then 200µl of microsphere suspension are injected in the chamber by the injection syringe pump, then the flow syringe pump is actuated. Microscope illumination is then turned on by the controller, and a movie with microspheres displacements and arrests is recorded. Then the illumination is turned off, and the whole cycle is repeated with a different shear rate. The operator turns off the automaton and movie acquisition when all shear conditions have been measured. The operator may then either disconnect the current chamber to connect one of the remaining eight, change the temperature, or change the set-up angle, then record a new movie and re-launch the automaton.
This method produce one initial movie containing several sequences (one for each shear rate), separated by non-illuminated (black) sequences. Post-processing is done by a plug-in written in

Java for ImageJ that cut the initial movie when illumination is off, get rid of non-illuminated sequences, and create separate movies for each shear stress. Theses sequences are then saved in a folder named after the initial movie, then particle detection and trajectory retrieving follows automatically.
